# Phase II trial of sorafenib and erlotinib in advanced pancreatic cancer

**DOI:** 10.1002/cam4.208

**Published:** 2014-02-12

**Authors:** Dana B Cardin, Laura Goff, Chung-I Li, Yu Shyr, Charles Winkler, Russell DeVore, Larry Schlabach, Melanie Holloway, Pam McClanahan, Krista Meyer, Julia Grigorieva, Jordan Berlin, Emily Chan

**Affiliations:** 1 Department of Medicine, Vanderbilt University Medical CenterNashville, Tennessee; 2 Department of Applied Mathematics, National Chiayi UniversityChiayi City, Taiwan; 3 Vanderbilt Center for Qualitative SciencesNashville, Tennessee; 4 Purchase Cancer GroupPaducah, Kentucky; 5 Tennessee Cancer SpecialistsKnoxville, Tennessee; 6 University Oncology/Hematology AssociatesChattanooga, Tennessee; 7 BiodesixBoulder, Colorado

**Keywords:** Erlotinib, pancreatic cancer, sorafenib, targeted therapy

## Abstract

This trial was designed to assess efficacy and safety of erlotinib with sorafenib in the treatment of patients with advanced pancreatic adenocarcinoma. An exploratory correlative study analyzing pretreatment serum samples using a multivariate protein mass spectrometry-based test (VeriStrat®), previously shown to correlate with outcomes in lung cancer patients treated with erlotinib, was performed. Patients received sorafenib 400 mg daily along with erlotinib 150 mg daily with a primary endpoint of 8-week progression free survival (PFS) rate. Pretreatment serum sample analysis by VeriStrat was done blinded to clinical and outcome data; the endpoints were PFS and overall survival (OS). Difference between groups (by VeriStrat classification) was assessed using log-rank *P* values; hazard ratios (HR) were obtained from Cox proportional hazards model. Thirty-six patients received study drug and were included in the survival analysis. Eight-week PFS rate of 46% (95% confidence interval (CI): 0.32–0.67) did not meet the primary endpoint of a rate ≥70%. Thirty-two patients were included in the correlative analysis, and VeriStrat “Good” patients had superior PFS (HR = 0.18, 95% CI: 0.06–0.57; *P* = 0.001) and OS (HR = 0.31 95% CI: 0.13–0.77, *P* = 0.008) compared to VeriStrat “Poor” patients. Grade 3 toxicities of this regimen included fever, anemia, diarrhea, dehydration, rash, and altered liver function. This study did not meet the primary endpoint, and this combination will not be further pursued. In this small retrospective analysis, the proteomic classification was significantly associated with clinical outcomes and is being further evaluated in ongoing studies.

## Introduction

Pancreatic adenocarcinoma is one of the most lethal malignancies in humans, and is currently the fourth leading cause of cancer death in the United States 1. Standard cytotoxic agents alone or in combination have made only incremental improvements in survival, and the advent of targeted therapies holds the promise of new ways to treat this recalcitrant malignancy. Our knowledge of the molecular abnormalities that occur during malignant transformation has grown, and it is now widely understood that pancreas tumors harbor a number of common alterations in a variety of core pathways involved in DNA damage control, invasion, and cell signaling 2. From these genetic analyses, we can conclude that therapies that impact a single targetable gene are less likely to be effective in this tumor type than agents that may impact a broader array of pathways or disrupt signaling at key nodal points, and the challenge has been to decide which combinations of targeted agents may lead to effective growth inhibition or prevention of spread of tumor cells.

The epidermal growth factor receptor (EGFR) initiates a signal transduction cascade that leads to modulation of cellular functions through activation of a number of pathways, including the phosphatidylinositol 3-kinase (PI3K) and the mitogen-activated protein (MAP) kinase pathways. In the NCIC CTG PA.3 study, a phase III trial of erlotinib plus gemcitabine versus single agent gemcitabine, the combination of erlotinib, the oral small molecule inhibitor of EGFR, with gemcitabine showed modest improvements in survival of patients with advanced pancreatic adenocarcinoma, but the absolute benefit was small and left many to conclude that this pathway was either not essential to tumor promotion or spread or that compensatory pathways would quickly make inhibition less effective for a majority of patients 3. To date, no clear marker has been validated to help us select the patients who are most likely to benefit from EGFR pathway inhibition, and thus this treatment regimen has not been widely embraced in clinical practice.

Given the genetic heterogeneity seen in pancreatic cancers, it is intuitive to ask which agents may be combined effectively to overcome redundancy found in many common pathways of proliferation and cell growth. Sorafenib is an attractive drug to combine with other targeted agents as it can impact a variety of pathways and can be tolerable for a majority of patients.

The combination of sorafenib and erlotinib inhibits the critical MAP kinase pathway at two levels. In addition, sorafenib targets vascular endothelial growth factor (VEGF) receptors and there are both preclinical and clinical evidence suggesting that blockade of both EGFR and VEGF pathways simultaneously have potential for additive, if not synergistic effects 4–6. Hints that the combination of erlotinib and sorafenib may have clinical activity have been seen in preclinical models in which human lung and colon cancer cell lines were exposed to these two agents, and demonstrated synergistic growth inhibition and apoptosis 7. A phase I trial combining these agents in 17 patients with advanced solid tumors also showed that this combination was well-tolerated with activity seen in patients with advanced gastrointestinal tumors (Partial response seen in one patient each with cholangiocarcinoma, pancreatic islet cell carcinoma, and small bowel adenocarcinoma) 8. Given the preclinical and early phase clinical data, this phase II study was designed to assess whether the combination of sorafenib and erlotinib would improve the 8-week progression-free survival (PFS) rate, as compared to a historical benchmark control, in patients with advanced pancreatic adenocarcinoma, in either the first- or second-line setting. Progression-free survival was chosen as the primary endpoint because it has been observed in a number of settings that other targeted agents such as sorafenib may demonstrate benefit in terms of survival or disease stabilization in the absence of objective responses by Response Evaluation Criteria in Solid Tumors (RECIST) criteria, and tumors of the pancreas in general demonstrate a low response rate by most objective criteria. Secondary objectives include response rate, PFS at 4 months, and safety profile of the combination in this patient population.

Clinical improvements seen with the addition of targeted agents to standard chemotherapy in unselected populations might be augmented by identifying a group of patients who are more likely to benefit from the combination. Unfortunately, clinically relevant tissue biomarkers in pancreatic cancer are lacking. VeriStrat (Biodesix, Boulder, CO) is a pretreatment multivariate protein test utilizing matrix-assisted laser desorption/ionization time of flight mass spectrometry that assigns a binary classification, VeriStrat Good (VS Good) and VeriStrat Poor (VS Poor), to serum or plasma samples. The test is based on a classification algorithm, utilizing eight distinct mass spectral features, comparing spectra from a patient's sample with a reference set identified in the study of clinical outcomes in patients with non-small cell lung cancer (NSCLC) treated with small molecule EGFR-tyrosine kinase inhibitors erlotinib and gefitinib 9. The test was independently validated in multiple retrospective studies of advanced NSCLC, metastatic breast cancer, colorectal cancer, and head and neck cancer that demonstrated that the test is prognostic and may predict for response to erlotinib, either alone or in combination in patients with NSCLC 10–13. In the present study, we carried out a retrospective analysis of pretreatment samples from advanced pancreatic cancer patients treated in the first- and second-line with erlotinib and sorafenib to evaluate the performance of the VeriStrat test with respect to PFS and overall survival (OS).

## Material and Methods

### Patient eligibility

Patients with a histologic diagnosis of advanced (locally advanced and unresectable or metastatic) pancreatic adenocarcinoma who received no more than one prior systemic therapy for advanced disease were eligible for this trial. Additional eligibility criteria included measurable disease by RECIST 1.0 criteria; age ≥18; Eastern Cooperative Oncology Group (ECOG) performance status of 0–2; adequate bone marrow, hepatic and renal function as defined by absolute neutrophil count (ANC) of ≥1500/mm^3^, platelet count ≥100,000/mm^3^, total bilirubin ≤1.5× the upper limit of normal (ULN), alanine aminotransferase (ALT) and aspartate aminotransferase (AST) ≤ 2.5× the ULN (or ≤5× the ULN for patients with liver involvement), and creatinine ≤1.5× ULN. Coagulation parameters were required to be within normal limits for patients not on chronic anticoagulation; patients on warfarin or heparin were allowed to participate but with close monitoring. Prior therapy with any antiangiogenic therapy was prohibited, as were patients with significant cardiac disease: congestive heart failure > class II (by New York Heart Association scale), unstable angina or new onset angina within the prior 3 months, or myocardial infarction within the prior 6 months. Other key exclusion criteria included known brain metastases, cardiac arrhythmias requiring antiarrhythmic therapy, uncontrolled hypertension despite optimal medical management, known human immunodeficiency virus (HIV), chronic hepatitis B or C, clinically significant infection, prior arterial thrombotic or embolic events in the prior 6 months, any significant bleeding event within 4 weeks of first dose of study drug, inability to swallow pills, or chronic untreated malabsorptive symptoms. All patients gave written informed consent in accordance with federal and institutional guidelines before study treatment.

### Treatment

Sorafenib was supplied by Bayer/Onyx Pharmaceuticals (Berlin, Germany), and erlotinib was provided by Astellas (Northbrook, IL). Treatment consisted initially of sorafenib 400 mg orally twice a day along with erlotinib 150 mg orally once a day, and 28 days of therapy were considered one cycle. Twelve of the first 15 patients required dose reduction early in therapy or had dose delays for toxicity, and the protocol was subsequently amended to begin with sorafenib 400 mg once a day with the option of escalation to twice daily dosing at the discretion of the treating physician if tolerable. Antiemetics were used onneed basis at the discretion of the treating physician and standard measures were used to deal with common skin toxicities of these regimens. Treatment was administered until disease progression, significant intercurrent illness, unacceptable adverse event(s), withdrawal of consent, noncompliance, need to stop therapy for toxicity for more than 28 days, or at the discretion of the treating physician. Doses of both drugs were modified as needed according to study guidelines.

### Assessments, follow-up, and monitoring

Patients were evaluated for response and disease progression by computed tomography (CT) every 8 weeks while on study, along with assessment of tumor marker (CA 19-9). Additional blood and serum for correlative assessments were obtained pretreatment and at 8 weeks. Toxicity assessments were performed by phone on weeks 2 and 6 after start of therapy, and in person at 4 weeks and every 4 weeks thereafter. Adverse events were graded according to the National Cancer Institute Common Terminology Criteria of Adverse Events (CTCAE v.3) and disease response by RECIST 1.0. Patients were monitored for adverse events from the time consent was signed and for efficacy once they began protocol therapy.

### Statistical methods

The primary objective of this study was to determine the 8-week PFS rate. The study was designed to detect a 70% 8-week PFS rate if it exists. The 8-week PFS rate is estimated by Kaplan–Meier method and its 95% confidence interval (CI) by Greenwood formula was reported. A sample size of 37 would achieve at least 80% power to detect a PFS difference of 4 weeks between the study arm and historical control data at a two-sided 0.1 significance level (type I error). This calculation was based on the one-sample exponential test. One patient who was eligible and had study drug distributed grew ill and never started study drugs and was not included in this analysis. Safety analyses included all patients who received at least one dose of therapy and included summaries of adverse events, serious adverse events, and adverse events leading to discontinuation of therapy.

### Proteomic test

Serum samples were collected within 14 days before the start of treatment. The samples were analyzed retrospectively by Biodesix using their standard VeriStrat test in a fully blinded fashion 11; the classification results were sent to the principal investigator. The statistical analysis was carried out after unblinding the clinical outcomes after receipt of VeriStrat classification. Statistical significance of difference in OS and PFS between groups (VeriStrat “Good” and VeriStrat “Poor”) was assessed using log-rank *P*-values. The hazard ratios (HRs) were calculated using Cox proportional hazard model. Analyses were performed using PRISM (Graphpad, La Jolla, CA) and SAS Enterprise Guide 4.3 (SAS, Cary, NC).

## Results

### Patient population

Between October 2008 and February 2011, 38 patients were accrued, with 36 included in the survival analysis. One patient was a screen failure, and one patient died prior to receiving protocol therapy. The majority of patients had an ECOG performance status at the start of therapy of 0 or 1 (92%). Thirty-four of the 37 subjects had received one line of prior therapy for advanced disease. Eleven patients were locally advanced, and 26 were metastatic at start of therapy. Thirty-two patients had adequate serum samples for inclusion into exploratory analyses by VeriStrat. Patient characteristics are summarized in Table 1.

**Table 1:** Demographic and baseline disease characteristics

Age, years	
Median	71
Range	40–81
Gender	
Female	18
Male	19
ECOG performance status	
0	9
1	24
2	4
Number of lines of prior therapy for advanced disease	
0	3
1	34
Stage at start of therapy	
Locally advanced	11
Metastatic	26

### Efficacy

**Figure 1:**
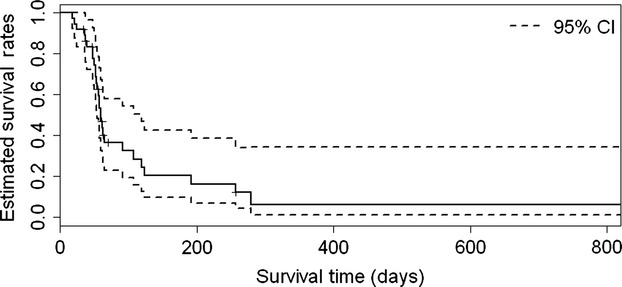
The 8-week progression-free survival rate is 46% (95% CI: 0.32–0.67). In order to achieve statistical significance, an 8-week progression-free survival rate ≥70% was needed.

The combination of sorafenib plus erlotinib did not improve either survival or PFS rate as compared to a historical control in this population. Eight-week PFS rate observed was 46% (95% CI: 0.32–0.67), which did not meet the goal PFS of ≥70%. (Fig. 1) Median OS was 99.5 days (95% CI: 71–188) or ∼3.3 months. Four-month PFS rate was 16.7% (95% CI: 0.08–0.346). Disease control rate was 24% (stable disease) at 8 weeks.

### Safety and tolerability

Treatment-related adverse events are summarized in Table 2. Twelve of the first 15 patients required dose delays or reductions for toxicity, and the protocol was subsequently amended to begin with sorafenib 400 mg once a day with the option of escalation to twice daily dosing at the discretion of the treating physician. Prior to this amendment, 20% of patients were able to stay on therapy with no dose modifications or reductions; after the amendment, 36% of patients were able to stay on therapy with no dose modifications or reductions. After this amendment, five patients required dose reductions, though this did not impact the need for dose delays substantially. In total, 11 patients (31%) required dose reductions: four had reductions in only sorafenib, four had reductions only in erlotinib, and three required reductions in both drugs. There were 21 instances of dose delays in 22 patients (59%): three attributed to sorafenib, three attributed to erlotinib, and 15 attributed to the combination of drugs. After the amendment starting sorafenib at a lower dose and allowing for escalation, two patients (9%) were able to escalate to 400 mg twice a day of sorafenib, and they eventually required dose delays, but no reductions. Most common adverse events related to treatment (frequency of >10%) were fatigue, rash, diarrhea, and abnormalities in liver function tests (transaminases, alkaline phosphatase, and bilirubin). One instance each of grade 4 rash and bilirubin were observed, and one patient developed acute hypoxia that was progressive and subsequently died, judged by the treating physician to be possibly related to study drugs. No unanticipated toxicities were encountered and overall therapy appeared to be more tolerable after amending the protocol.

**Table 2:** Grades 3–5 treatment-related adverse events

Hematologic	
Lymphopenia	2
Anemia	2
Thrombocytopenia	1
Nonhematologic	
Hypertension	3
Fever	1
Fatigue	5
Rash	3*
Dehydration	2
Diarrhea	4
Nausea	1
Vomiting	1
Anorexia	2
Transaminases/amylase	4
Alkaline phosphatase/bilirubin	3*
Hypophosphatemia	2
Neuropathy	1
Abdominal pain	2
Pulmonary	1*
Thrombosis	2

* One grade 4 rash and hyperbilirubinemia each observed; one grade 5 hypoxia observed.

### Proteomic analysis results

Thirty-two patients were included in the final analysis. Of the samples that were received by Biodesix, two were excluded due to hemolysis, 27 classified as VS Good, nine as VS Poor, and one Indeterminate (excluded from the analysis); two VS Good and two VS Poor samples were not used in the survival analysis because of the absence of clinical outcome data. Of the 32 patients evaluable for response, no partial responses were observed in either VS group; stable disease was observed only in seven patients with VS Good classification, and 10 and five patients had progressive disease in VS Good and VS Poor groups, respectively (*χ*^2^ *P* = 0.23).

**Figure 2:**
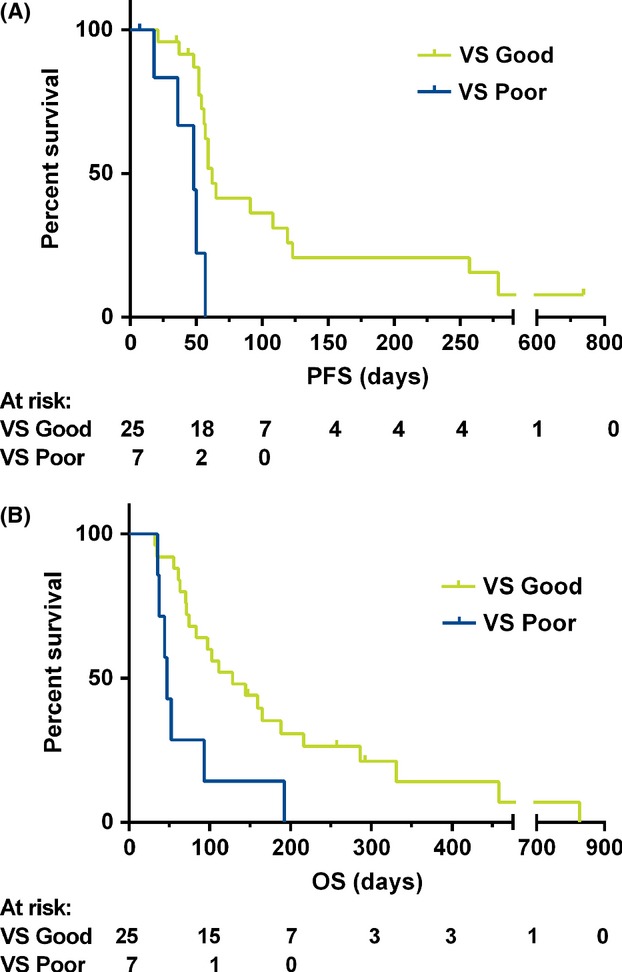
Kaplan–Meier plots of PFS (A) and OS (B) grouped by VeriStrat Classification

Patients with a pretreatment VS Good classification had statistically significantly longer PFS and OS compared to those classified as VS Poor (Fig. 2A and B): median PFS was 62 days (2.1 months) in the VS Good group and 48 days (1.6 months) in the VS Poor group, the hazard ratio (HR) between groups was 0.18 (95% CI: 0.06–0.57), with *P* = 0.001. For OS, the HR was 0.31 (95% CI: 0.13–0.77), *P* = 0.008, median OS 128 days (4.3 months) and 47 days (1.6 months) in the VS Good and VS Poor groups, respectively (Table 3).

**Table 3:** Summary of outcomes by proteomic classification

	Median survival (days)		*P*-value	HR (95% CI)
	
	“Good” (*n* = 25)	“Poor” (*n* = 7)		
PFS	62	48	0.0011	0.18 (0.06–0.57)
OS	128	47	0.0078	0.31 (0.13–0.77)

## Discussion

Effective therapies are urgently needed for the treatment of advanced pancreatic cancer. While recent combinations of cytotoxic agents such as FOLFIRINOX 14 and gemcitabine plus nab-paclitaxel 15 have made modest improvements in the survival of patients with advanced disease, the toxicity of these combination therapies can be prohibitive and only a subset of patients are candidates for this type of therapy. Moreover, targeting the underlying mechanisms of pancreatic cancer with more specific agents will likely be essential to substantially improve the survival of patients with this disease as standard cytotoxic agents have shown limited benefit. Patients who are able to tolerate aggressive cytotoxic regimens in the first-line setting may also need or desire less toxic therapies when they transit to later lines of therapy, so more agents and more tolerable agents are especially needed. This study testing combination-targeted agents without chemotherapy in the second-line setting demonstrates that this population can be recruited to clinical trials. The combination of erlotinib plus sorafenib as either first or second-line therapy was attractive from both a mechanistic standpoint, as well as a logistical one, as this was a study of two oral agents requiring less frequent travel for drug administration.

Unfortunately, with an 8-week PFS rate of 46%, the results of this study failed to meet prespecified criteria for success (i.e., 8-week PFS of 70%) for the use of sorafenib and erlotinib in the treatment of unselected patients in mostly the second-line setting. There are few good comparator studies for second-line therapy, but a randomized phase III study comparing a regimen of oxaliplatin, folinic acid, and fluorouracil to best supportive care (BSC) in patients who received first-line gemcitabine demonstrated a median survival time of 4.8 months for the active arm, compared to 2.3 months for the BSC arm 16. The study had to be closed early due to low accrual likely due to diminishing acceptance of BSC in the second-line setting for this patient population and was amended to make the comparator arm fluorouracil plus folinic acid. The final results of this larger study of 165 patients demonstrated a median OS of 28 weeks (vs. 13 for the flurouracil arm) 17. While not directly comparable, the median OS in our study was 3.3 months, which is shorter than the chemotherapy arm of the prior study, but longer than the BSC arm, and not as low as would be anticipated given the fairly low 8-week PFS rate. This may have been slightly skewed by a small cohort of patients (7) who had a survival of ≥6 months (range of 6–20 months) after coming off study. Eleven patients had nonmetastatic disease at the start of therapy and six of these patients made up part of this long-lived group.

There have been a few small clinical studies investigating the role of sorafenib in pancreatic cancer. Gemcitabine plus sorafenib was tested in advanced solid tumors with an expanded cohort of pancreatic cancers, with over half of the pancreatic cancer patients achieving stable disease, and with a median PFS of 108 days for this cohort 18. However, a small phase II trial conducted at the University of Chicago combining gemcitabine with sorafenib in patients with unresectable pancreatic cancer failed to meet its primary endpoint of overall response rate at interval analysis and was closed to further accrual 19.

Given that patients with localized disease in this study still had very heterogeneous survival, there is a need to identify factors that could predict improved outcomes with therapy. Erlotinib has been previously shown to impact survival in patients with pancreatic cancer when combined with gemcitabine, though the overall magnitude of benefit was small, suggesting a subpopulation of patients may derive most of the benefit, possibly due to intrinsic differences in molecular subtypes or other tumor characteristics. Evidence of such subtypes was shown by Dr. Collisson and colleagues, correlating clinical patient data with laboratory models of these distinct subtypes, and their findings confirm that KRAS mutation status is not an ideal predictor of response to EGFR inhibition 20. The VeriStrat assay is a pretreatment blood-based test that has been shown to correlate with outcomes after EGFR-tyrosine kinase inhibitor therapy in NSCLC patients 9. The test has been shown to be both prognostic of better or worse PFS and OS in the absence of therapy 10 and predictive of differential survival benefit from erlotinib versus chemotherapy in the second-line NSCLC patients 21: patients classified as VS Poor have worse prognosis and benefit more in terms of OS from chemotherapy rather than from erlotinib, as compared to VS Good. For the sorafenib–erlotinib combination in advanced NSCLC patients, significant separation between patients with VS Good and VS Poor classifications for PFS and OS have been demonstrated 22. The recently completed prospective randomized proteomic-stratified phase III study of second-line erlotinib versus chemotherapy in patients with inoperable NSCLC trial (PROSE) has convincingly demonstrated that VeriStrat is predictive of differential benefit from chemotherapy or erlotinib in OS depending on test classification (the *P*-value of VeriStrat by treatment interaction 0.031) 21. However, in the retrospective analysis of samples from pancreatic patients treated with gemcitabine or a combination of gemcitabine with erlotinib in the PA.3 study, the relative advantage of VS Good over VS Poor patients was similar in both treatment arms 23.

In this study, the VS Good classification, compared to VS Poor, was statistically significantly associated with longer PFS (62 vs. 48 days, HR = 0.18, *P* = 0.001) and OS (128 vs. 47 days, HR = 0.31, *P* = 0.008). Also of note is that stable disease was observed only in the patients with the VS Good classification. While the numbers are quite small, and there is no comparator arm on the study, this is a provocative result and further evaluation is warranted. The previously published results of the PA.3 study on the combination of gemcitabine plus erlotinib did not show an improvement in patient survival compared to gemcitabine alone by what many feel is a clinically meaningful margin. In the retrospective analysis of samples from the PA.3 study, VeriStrat was significantly associated with OS and PFS in both treatment arms. The relative PFS and OS advantage of VS Good over VS Poor classification was similar in both treatment arms, with no significant interaction, suggesting that VeriStrat in this setting may not be predictive of response to erlotinib but rather prognostic in patients with pancreatic cancer 23. Given the conflicting results across studies, it remains unclear at this time whether the VeriStrat test is merely selecting out a better prognosis group of patients in pancreatic cancer or if it may have utility in selecting patients likely to respond to a particular regimen. To further investigate, we have incorporated the collection of serum samples into an ongoing study at our institution that combines gemcitabine, erlotinib, and dasatinib for patients with advanced pancreatic cancer. (NCT01660971) Hopefully, continued efforts to understand which patient will respond to a particular therapy will be a foundation to develop more effective, personalized regimens.
